# Estimating the risk of post-COVID condition in deprived communities, migrants and ethnic minorities in England: findings from Virus Watch—a prospective community cohort study

**DOI:** 10.1136/jech-2024-223491

**Published:** 2025-07-07

**Authors:** Wing Lam Erica Fong, Sarah Beale, Vincent Nguyen, Jana Kovar, Alexei Yavlinsky, Andrew C Hayward, Ibrahim Abubakar, Sander MJ van Kuijk, Robert Aldridge

**Affiliations:** 1 Institute of Health Informatics, University College London, London, UK; 2 Department of Clinical Epidemiology and Medical Technology Assessment, Maastricht University Medical Centre (MUMC), Maastricht, The Netherlands; 3 Institute of Epidemiology and Health Care, University College London, London, UK; 4 Department of Population, Policy and Practice, University College London Great Ormond Street Institute of Child Health, London, UK; 5 Faculty of Population Health, University College London, London, UK; 6 The Institute for Health Metrics and Evaluation, University of Washington, Seattle, Washington, USA

**Keywords:** COVID-19, EPIDEMIOLOGY, Health inequalities

## Abstract

**Background:**

Deprived communities, migrants and ethnic minorities were disproportionately affected by COVID-19 and may, therefore, be at a higher risk of post-COVID condition (PCC). This analysis, using data from the Virus Watch study, investigates how deprivation, migration status and ethnic minority status influence PCC risk in both the full cohort (all regardless of infection status) and those with a confirmed COVID-19 infection.

**Methods:**

A subset of participants from Virus Watch, a prospective community cohort study in England, were included. We used logistic regression to compare the predicted probability of developing PCC in both full and infected cohorts among different deprivation levels, migration and ethnic minority status categories by sex-at-birth during pre-Omicron and Omicron periods, adjusting for sociodemographic covariates.

**Results:**

During the pre-Omicron period, PCC probability increased with deprivation levels, especially in females (most deprived: 7.8%, 95% CI 4.6% to 11.0%; least deprived: 3.5%, 2.5%–4.5%). Migrant and ethnic minority males had a higher likelihood of PCC than their respective counterparts, particularly in the full cohort for migrants (6.3%, 1.8%–10.8%) and the previously infected cohort for ethnic minorities (38.8%, 21.2%–56.4%). However, these disparities were less pronounced in females. In the Omicron period, these differential probabilities were also less evident.

**Conclusion:**

Our findings suggest that greater PCC probability among these populations is driven by increased infection risk and postinfection determinants. This underscores the need for policies and interventions to reduce infection risk and affordable and easily available healthcare services for those with PCC.

**WHAT IS ALREADY KNOWN ON THIS TOPIC**

- Deprived, migrant and ethnic minority groups experienced disproportionate rates of SARS-CoV-2 infection, hospitalisation and death due to a range of socioeconomic factors.
- Emerging evidence suggests that individuals living in deprived areas may have a higher risk of developing post-COVID condition (PCC), while mixed results were reported in research on PCC risk in ethnic minorities.
- There is limited evidence on the association between migration status and PCC risk.

**WHAT THIS STUDY ADDS**

- During the pre-Omicron period, more deprived populations were associated with an increased PCC probability, especially among females. Migrant, ethnic minority and migrant-ethnic minority males also had higher PCC probabilities compared with their counterparts after accounting for potential confounders.
- In the Omicron period, the disparities in the likelihood of PCC were less pronounced, though migrant-ethnic minority females still had higher PCC probability, especially in those with confirmed infections, suggesting postinfection determinants play a role.
- Our study suggests preinfection and postinfection determinants of PCC among these populations.

**HOW THIS STUDY MIGHT AFFECT RESEARCH, PRACTICE OR POLICY**

- This study underscores the need to address health inequalities faced by deprived, migrant and ethnic minority populations to reduce their risk of COVID-19 infection and PCC.
- Further research into the intersection of migration, ethnicity and deprivation, along with other factors such as sex and occupation, is required to guide targeted public health efforts and resource allocation.
- Targeted policies should address occupational and housing exposure risks, increase vaccine uptake, provide culturally appropriate public health information and ensure equitable access to PCC and primary care services.

## Introduction

 Migrants, ethnic minorities and individuals in deprived areas have historically experienced health inequalities which were exacerbated by the COVID-19 pandemic.^1^ The pandemic also led to the emergence of post-COVID condition (PCC) or long COVID, a syndrome comprising a wide range of symptoms which emerge within 3 months of acute infection and persist for at least 2 months with no other explanation. Globally, 10%–30% of non-hospitalised and 50%–70% of hospitalised cases reported developing PCC.^2^

Known PCC risk factors include increasing age, female sex, and severe COVID-19 illness, but the role of socioeconomic factors remains unclear.^2^ Deprived communities, migrants and ethnic minorities experienced higher rates of SARS-CoV-2 infection, hospitalisation and death compared with the general population, particularly during the Wild-type, Alpha, and Delta variant waves.^3^,^5^ Socioeconomic disparities, including household overcrowding, occupational exposures, and limited healthcare and vaccine access, contributed to these increased risks.^6^,^9^ These communities also have higher rates of underlying health conditions, such as diabetes and hypertension, which increase their vulnerability to severe COVID-19.^1^ As a result, they may be at greater risk of developing PCC.

Emerging evidence suggests that individuals in deprived areas may be at an elevated risk of developing PCC. Multiple UK studies have consistently reported greater odds/risk of PCC in the most deprived decile/quintile compared with the least deprived after adjusting for covariates.^10^ However, studies on PCC risk in ethnic minorities yielded mixed results. Research from the UK, USA, the Netherlands and Denmark^11^,^15^ observed higher PCC odds/risk among ethnic minorities compared with the majority ethnic population. In contrast, others reported no association^16 17^ or lower PCC odds/risk.^18 19^ Evidence on migration status is limited, but a study in the Netherlands found higher PCC risk in migrant hospitalised patients compared with non-migrants following confounder adjustment.^12^

Given these inconsistencies and gaps in research, we aimed to use data from the Virus Watch prospective community cohort study in England^20^ to investigate how PCC risk varies by deprivation levels, migration and ethnic minority status during pre-Omicron and Omicron periods. We evaluated this in the full cohort, irrespective of infection status (objective 1), and in individuals with confirmed COVID-19 infection (objective 2). This will provide evidence for policies addressing health inequities related to PCC and future pandemics.

## Methods

### Ethics approval and consent

Virus Watch was approved by the Hampstead NHS Health Research Authority Ethics Committee: 20/HRA/2320, and conformed to the ethical standards set out in the Declaration of Helsinki. Participants provided informed consent for all aspects of the study.

### Study design and participants

Virus Watch is a large prospective community cohort study established in June 2020 on the transmission and burden of COVID-19 in England and Wales (n=58 497 as of March 2022). Participation was self-selected and limited to households with internet access and a lead household member proficient in English (Consent forms and participant information sheets were available in multiple languages). Participants completed weekly online questionnaires regarding COVID-19 symptoms, testing and vaccination and occasional in-depth surveys on COVID-19 related topics, such as healthcare access, behavioural practices and long-term symptoms. The full study design and methodology have been described elsewhere.^21^ We followed the Strengthening the Reporting of Observational Studies in Epidemiology guidelines for reporting our observational study.^22^

Participants in this analysis were a subset of the Virus Watch study cohort. Participants were included if they (1) were registered with an English postcode, (2) responded to at least one survey on new persistent symptoms between February 2020 and March 2023 and (3) if infected, had an assignable infection variant based on their SARS-CoV-2 test reporting date (online supplemental table 2). Welsh participants were excluded due to limitations in data on variant periods.

### Exposures

We examined three exposures: Index of Multiple Deprivation (IMD) as a measure of deprivation, migration status and ethnic minority status.

IMD is calculated for each small local area in England across seven dimensions of deprivation, including crime, employment, education, income, health, living environment and barriers to housing and services. Areas are then ranked from most to least deprived relative to other areas and divided into five equal groups, with the first quintile representing the most deprived and the fifth representing the least deprived.^23^ IMD was assigned based on self-reported postcode of residence, which was linked to the May 2020 ONS Postcode Lookup.^23^ The fifth quintile was used as the reference category.

Migration status was determined by self-reported country of birth. Non-UK-born participants were classified as migrants, while those with missing migration status were analysed as a separate category. Comparisons used the UK-born group as the reference category.

Ethnic minority status was based on self-reported ethnicity. Participants who reported to be white Irish, white Other, mixed, South Asian, other Asian, Black or other were categorised as ethnic minorities. The white British group acted as the reference category.

### Outcome

PCC (yes/no) was phenotypically defined using the WHO consensus definition as the onset of at least one symptom lasting at least two months within three months of acute SARS-CoV-2 infection, unexplained by other diagnoses. SARS-CoV-2 infections before the end of nationally funded COVID-19 testing (31 March 2022) were identified using linked test results from the national testing database, self-reported/study-based COVID-19 and antibody test results (see ‘Sources of SARS-CoV-2 Infection’ in online supplemental materials).

Persistent symptom tracking was based on four in-depth surveys on long-term symptoms that were administered within three years (February 2021, May 2021, March 2022 and March 2023), where participants reported any symptoms lasting at least four weeks, the onset dates of their three most severe symptoms and whether they were ongoing or resolved. Data were available from February 2020 to March 2023, with symptom onset dates matched to track symptom episodes across multiple surveys.

PCC cases were defined as individuals with a confirmed SARS-CoV-2 infection within three months of symptom onset with at least one symptom lasting two months. Symptom duration was calculated from symptom onset to survey completion (for ongoing symptoms) or reported resolution date. Only PCC cases reported by 30 June 2022 (three months post nationally funded testing end) were included to minimise misclassification. Participants who were hospitalised for/with COVID-19 (n=55) or reported ongoing symptoms lasting less than two months (n=47) were also excluded to minimise the risk of misclassifying acute COVID-19, Post-Intensive Care Syndrome or Post-Sepsis Syndrome as PCC cases.^24^,^26^ Non-PCC participants completed all four surveys and either reported no persistent symptoms or symptoms without a confirmed SARS-CoV-2 infection within three months of onset, reducing the risk of undetected or unreported symptoms.

### Covariates

The covariates considered were age group (<25, 25–44, 45–64 and 65+years), sex at birth (male and female) and occupation. Occupations were categorised into five categories based on occupation type, exposure risk and employment status: higher exposure risk occupation, lower exposure risk occupation, not in employment, retired and unknown/other status (see online supplemental table 1 and further details elsewhere).^27^

SARS-CoV-2 infection variants were based on regional variant dominance periods.^28 29^ Study periods were categorised as ‘pre-Omicron’ (1 February 2020–13 December 2021) and ‘Omicron’ (14 December 2021–12 June 2022) (online supplemental table 2).

### Statistical analyses

We summarised baseline sociodemographic and clinical characteristics using descriptive statistics.

Separate analyses were conducted for each exposure using two distinct cohorts. The first included all participants, regardless of SARS-CoV-2 infection history, to assess the association between each exposure and PCC. The second included individuals with a confirmed SARS-CoV-2 infection to estimate the association between each exposure and the risk of PCC within infected populations, providing an estimate of postinfection PCC probability differences among different exposure groups.

Directed acyclic graphs (DAGs) were used to identify minimally sufficient adjustment sets for each exposure (online supplemental figures 1–3). For IMD quintile, the adjustment set included age, migration status, ethnic minority status and occupation for objective 1, and age, migration and ethnic minority status for objective 2. For migration status, the set included age and minority ethnicity status for both objectives, while for minority ethnicity status, the adjustment set consisted of age. BMI and clinical vulnerability were considered due to their influence on acute infections and the development of long-term symptoms.^30 31^ However, they were not included in the adjustment sets since they were deemed as mediator variables in the DAGs.

We conducted stratified multivariable logistic regression analyses for each exposure and cohort, stratified by sex and variant period. Non-UK-born ethnic minorities often face unique risk factors and intersecting social determinants that may affect their health outcomes.^32^ Therefore, to explore the intersection of minority ethnicity and migration status on the likelihood of developing PCC, we performed an additional analysis using a composite variable that combined these two factors, adjusted for age.^33^ Furthermore, a sensitivity analysis was conducted by categorising white British participants with missing migration status as UK-born, based on evidence from the 2011 Census that 98% of white British individuals were UK-born.^33^ Multicollinearity was tested (see ‘Multicollinearity’ in online supplemental materials). Results were expressed as predicted probabilities (PP) to allow comparison across all exposures, and odd ratios (OR) to assess the relative odds of developing PCC in each exposure group compared with the reference group in both cohorts.

## Results

**Figure 1:**
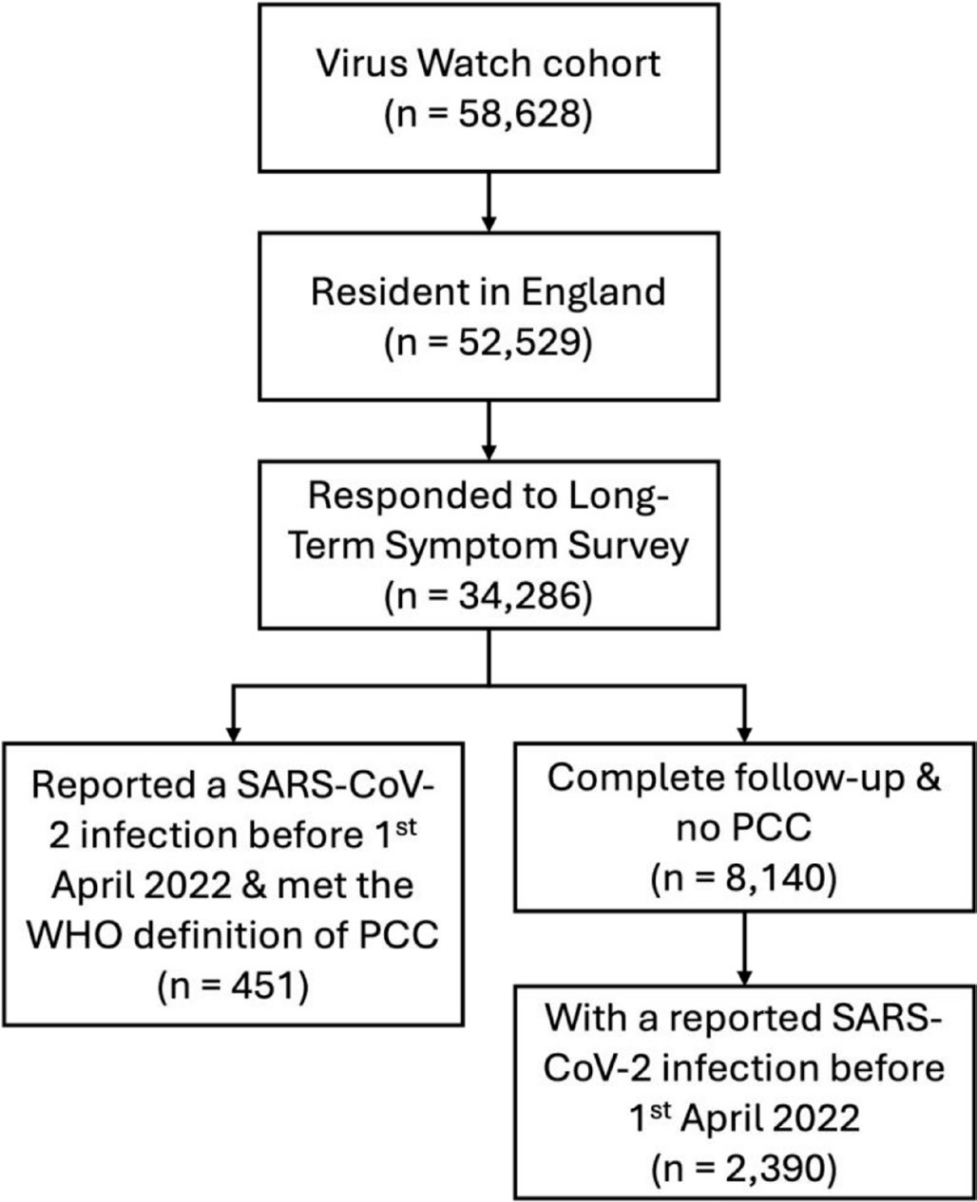
Flow diagram of eligibility. PCC, post-COVID condition.

Figure 1 illustrates participant selection. Table 1 reports the sociodemographic and clinical characteristics of the analysis cohorts. Compared with the population in England, our study cohort tended to be older, female, White British, UK-born and residing in less deprived areas (table 1). See online supplemental figures tables 6–8 for characteristics by each exposure.

**Table 1:** Demographic and clinical characteristics of the analysis cohorts compared with the population of England (from the Office for National Statistics (ONS))

Characteristic	All participants regardless of any confirmed SARS-CoV-2 infection n=8591	Participants with a reported confirmed SARS-CoV-2 infection n=2841	Population of England from ONS^44 45^ (%)
Age group			
0–24	415 (4.8%)	231 (8.1%)	29
25–44	477 (5.6%)	267 (9.4%)	26
45–64	3185 (37%)	1141 (40%)	26
65+	4514 (52%)	1202 (42%)	19
Sex (at birth)			
Female	4906 (57%)	1655 (58%)	51
Male	3685 (43%)	1186 (42%)	49
Ethnic minority status			
White British	7870 (92%)	2578 (91%)	74
Minority ethnic	666 (7.8%)	250 (8.8%)	26
Missing/prefer not to say	55 (0.6%)	13 (0.5%)	–
Migration status			
UK-born	7299 (85%)	2355 (83%)	83
Not UK-born	537 (6.3%)	171 (6.0%)	17
Missing	755 (8.8%)	315 (11%)	–
IMD quintile			
First quintile (most deprived)	593 (6.9%)	213 (7.5%)	20
Second	1139 (13%)	406 (14%)	20
Third	1734 (20%)	573 (20%)	20
Fourth	2274 (26%)	714 (25%)	20
Fifth quintile (least deprived)	2778 (32%)	921 (32%)	20
Missing	73 (0.8%)	14 (0.5%)	–
Clinical vulnerability			
Not clinically vulnerable	4856 (57%)	1679 (59%)	–
Clinically vulnerable	2391 (28%)	763 (27%)	–
Clinically extremely vulnerable	1262 (15%)	370 (13%)	–
Missing	82 (1.0%)	29 (1.0%)	–
Body Mass Index (BMI)			
Underweight	82 (1.0%)	24 (0.8%)	–
Normal	2836 (33%)	898 (32%)	–
Preobesity	2535 (30%)	808 (28%)	–
Obesity	1361 (16%)	459 (16%)	–
Severe obesity	174 (2.0%)	48 (1.7%)	–
Missing	1603 (19%)	604 (21%)	–
Occupation			
Higher risk occupation	1386 (16%)	591 (21%)	–
Lower risk occupation	1746 (20%)	643 (23%)	–
Not in employment	358 (4.2%)	118 (4.2%)	–
Retired	4120 (48%)	1105 (39%)	–
Unknown or other status	981 (11%)	384 (14%)	–

IMD, Index of Multiple Deprivation.

**Figure 2:**
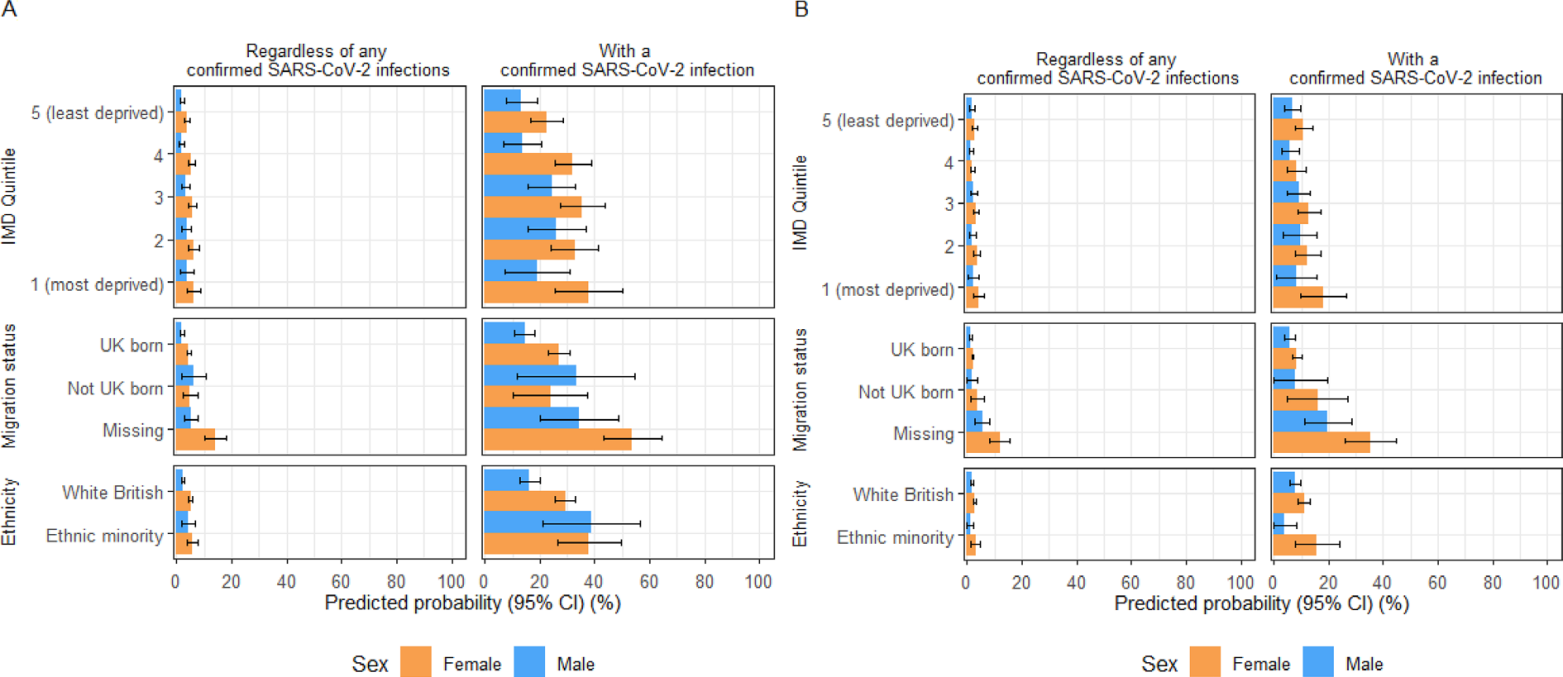
Predicted probabilities of PCC by IMD quintile, migration status and ethnic minority status in the full cohort and individuals with a previous SARS-CoV-2 infection, categorised by reported sex-at-birth during (**A**) pre-Omicron and (**B**) Omicron periods. IMD, Index of Multiple Deprivation; PCC, post-COVID condition.

Across all exposure groups, the likelihood of developing PCC was higher during the pre-Omicron than the Omicron period, regardless of sex and infection status. Overall, the PCC probability was higher in females than males across all exposure groups (figures2  3; online supplemental tables 9–12, 29, 30).

**Figure 3:**
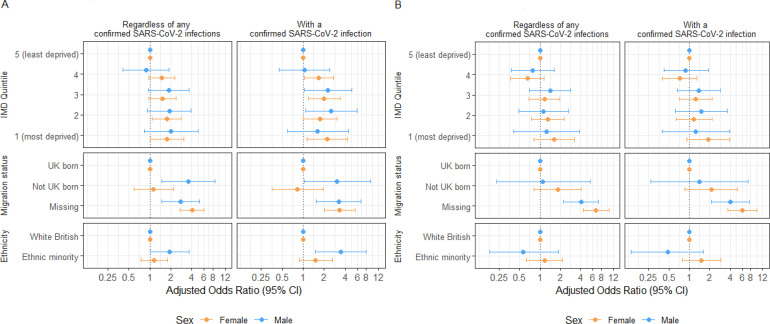
Conditional adjusted OR of PCC by IMD quintile, migration status and ethnic minority status in the full cohort and individuals with a previous SARS-CoV-2 infection, categorised by reported sex-at-birth during (**A**) pre-Omicron and (**B**) Omicron periods. IMD, Index of Multiple Deprivation; OR, odds ratio; PCC, post-COVID condition.

### PCC risk by IMD quintile

Pre-Omicron, increasing PCC probability across deprivation levels was observed for both sexes, regardless of infection status (figure 2A). Among individuals with confirmed infection, females residing in the most deprived quintile and males in the second most deprived quintile had the highest PCC probabilities (female: 37.7%, 95% CI=25.4% to 50.0%; male: 26.1%, 15.5%–36.7%) (online supplemental table 11). There was strong evidence for increased odds of PCC in females in the most deprived areas (adjusted OR (aOR): 2.1, 1.1–4.3) and males in the second most deprived areas (2.5, 1.1–5.8) compared with counterparts in the least deprived areas (figure 3A; online supplemental tables 13 and 14).

In the Omicron period, there were no between-quintile differences for either sex in both cohorts (figure 2B). Females in the most deprived areas had the highest PCC probability (18.1%, 9.6%–26.6%), exceeding those in less deprived areas and all males (online supplemental table 12). However, there was little evidence of increased odds in more deprived groups than those least deprived in both cohorts during this period (figure 3B; online supplemental tables 15 and 16).

### PCC risk by migration status

Pre-Omicron, migrant males had a higher PCC probability compared with UK-born males in the full cohort (migrant: 6.3%, 1.8%–10.8%; UK-born: 1.9%, 1.4%–2.4%) and the confirmed infection cohort (migrant: 33.1%, 11.8%–54.5%; UK-born: 14.4%, 10.5%–18.3%) (figure 2A; online supplemental table 11). There was strong evidence for a 3.6 (1.5–8.5) increased odds of PCC in migrant males compared with UK-born males in the full cohort, but this was attenuated in the infected group (aOR: 3.1, 1.0–9.2) (figure 3A; online supplemental tables 17 and 18). Individuals with missing migration status had the highest PCC probabilities in both cohorts.

During the Omicron period, point estimates for PP of PCC tended to be higher for non-UK-born than UK-born individuals in both cohorts, but CIs overlapped (figure 2B; online supplemental table 12). There was also little evidence to suggest differential odds between the two groups (figure 3B). Similar to the pre-Omicron period, individuals with unknown migration status exhibited the highest PCC probability, particularly among females (female: 35.1%, 25.8%–44.5%; male: 19.8%, 11.2%–28.4%) (online supplemental tables 19 and 20).

Reclassifying white British participants with missing migration status as UK-born attenuated the increased PCC probability seen in non-UK-born males in both cohorts during the pre-Omicron period. The increased PCC risk among those with unknown migration status was also lost (online supplemental figure 4 amd tables 25–28).

### PCC risk by ethnic minority status

Pre-Omicron, ethnic minority males had a higher PCC probability than white British males in the full cohort, with ethnic minority males experiencing a 1.93 (1.01–3.68) higher odds of PCC. A similar trend was seen in the infected cohort (white British: 38.8%, 21.2%–56.4%; ethnic minority: 16.2%, 12.5%–20.0%), with the association observed being more pronounced (aOR: 3.44, 1.49–7.94). Among females, we found no evidence of an association between ethnic minority status and PCC in both cohorts (figures2A  3A; online supplemental tables 11; 21 and 22).

In the Omicron period, PCC probability was generally low across all groups, regardless of infection status. Among infected individuals, the likelihood of PCC was higher in ethnic minority than white British females (white British: 11.0%, 9.0%–13.0%; ethnic minority: 15.7%, 7.5%–23.8%) but lower in ethnic minority males than white British males (white British: 7.8%, 5.8%–9.7%; ethnic minority: 3.9%, 0.0%–8.3%), though CIs overlapped (figure 2B; online supplemental table 12). AORs suggested no differences in the odds of developing PCC between ethnic groups for either cohort and sex (figure 3B; online supplemental tables 23 and 24).

**Figure 4:**
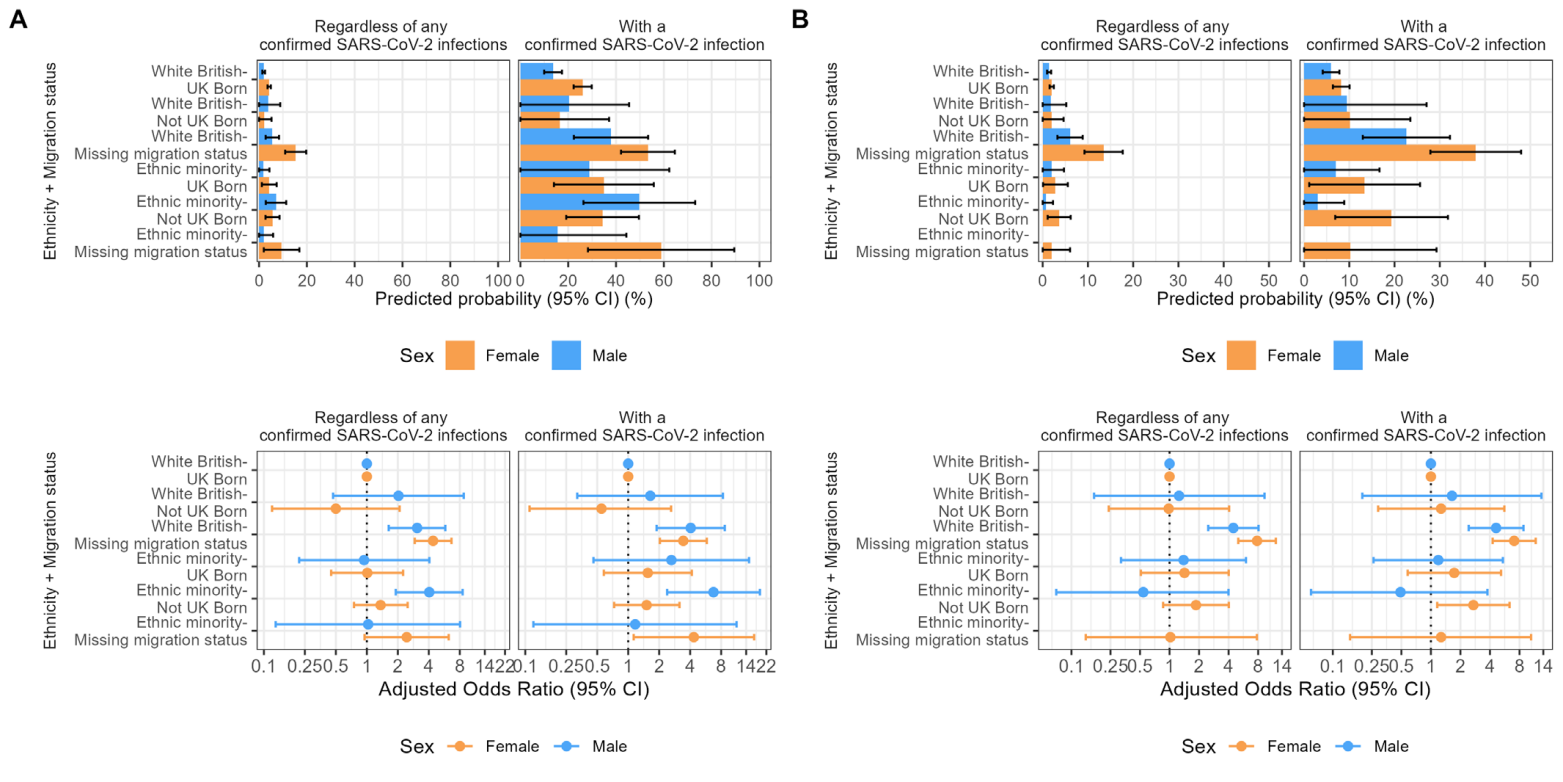
Predicted probability and conditional adjusted OR of PCC by the ‘ethnicity-migration’ composite variable in the full cohort and individuals with a previous SARS-CoV-2 infection, stratified by reported sex-at-birth during (**A**) pre-Omicron and (**B**) Omicron periods. No PP and OR estimates are available for males in the ‘ethnic minority-missing migration status’ group from the Omicron period due to the small sample size. PCC, post-COVID condition; OR, odds ratio; PP, predicted probabilities.

When including the ‘ethnicity-migration status’ composite variable, among the infected cohort, we found strong evidence for a higher PCC probability in male ethnic minority migrants (49.7%, 26.4%–73.1%) compared with white British UK-born males (13.7%, 9.9%–17.4%) during the pre-Omicron period, and in female, ethnic minority migrants (19.4%, 6.9%–31.8%) than their white British UK-born counterparts (8.2%, 6.4%–10.1%) during the Omicron period (figure 4; online supplemental tables 29–34).

## Discussion

Using data from a community cohort in England, we found that deprivation levels were associated with higher PCC probability, particularly among females, during the pre-Omicron period. Migrant and ethnic minority groups had higher probabilities of PCC, though these disparities were less pronounced in females—who overall had a higher probability of PCC—and more pronounced in migrant-ethnic minority males. These disparities were also less pronounced in the Omicron period, with some evidence for an association with PCC for migrant-ethnic minority females than their white British-UK-born counterparts.

While our study lacked statistical power to demonstrate an association between deprivation and PCC risk, the point estimates suggested one, which is consistent with broader literature. Shabnam *et al* found higher PCC risk in females than males across all IMD deciles and a dose–response relationship with deprivation levels in both sexes.^10^ Our findings regarding elevated PCC probability among ethnic minority males align with some existing studies, although findings on ethnic disparities have been mixed.^11^,^19^ Furthermore, our results corroborate those from the Netherlands, which reported higher PCC risk in migrants.^12^ It is important to note that our findings are not directly comparable since our analyses were stratified by sex and variant period. Our findings underscore the intersectionality of sex, ethnicity, migration and deprivation as PCC determinants and the need for further comparative research to better understand these intersections.

Our findings suggest that inequalities in the likelihood of PCC were influenced by preinfection and postinfection determinants. Migrant and ethnic minority males, who are often employed in insecure or frontline roles, reliant on public transport, working or residing in overcrowded environments, and experiencing pre-existing health inequalities, likely faced heightened exposure risk.^6^,^9^ These findings may reflect intervention-induced inequalities during the pre-Omicron period—where measures including lockdowns, social distancing and work-from-home, while lowering overall infection rates, disproportionately impacted these populations depending on work and housing-related exposures. Differential vaccine access in these populations may also affect their infection risk.^34^ However, by the Omicron period, as restrictions were lifted, the observed inequality gaps narrowed when infection became more widespread.

Slower vaccine uptake among migrants with non-White ethnicity, along with lower vaccination rates in ethnic minority and deprived groups, as reported by Burns *et al* and Curtis *et al*, likely also contributed to the increased risk of infection and severe illness, particularly by pre-Omicron variants.^35^,^37^ These variants were associated with more severe COVID-19 illness, a key infection-related PCC determinant.^38^ Improved vaccination coverage across different populations and Omicron’s reduced pathogenicity contributed to milder illness and, subsequently, the observed attenuation of PCC risk disparities in the later period.^37 39^ This underscores the need to improve vaccine access and uptake in deprived and minority groups, particularly in future pandemics, to reduce their infection and PCC risk.

Delayed care for COVID-19, often due to institutional and personal barriers to healthcare access, may lead to more severe illness and increase the risk of PCC.^40^ The individual and intersecting characteristics of migrants and ethnic minorities also translate to unique challenges related to healthcare access, such as cultural and language differences, fear of stigma and distrust of healthcare systems.^40^ Job insecurity and lack of paid sick leave, common among deprived communities and in some migrant and ethnic minority groups, also delay or prevent timely care, as many cannot afford to miss work or risk losing their jobs.^40 41^ These challenges are more pronounced for women in these populations as they are more likely to work in low-income, precarious jobs and serve as primary caregivers.^42^

### Strengths and limitations

To the best of our knowledge, this is the first study to examine the association between migration status and PCC in England, assess how deprivation, migration and minority ethnicity status influence PCC risk over time during pre-Omicron and Omicron periods, and explore whether PCC determinants of these populations arise before or after a SARS-CoV-2 infection. Virus Watch’s longitudinal design and detailed sociodemographic and clinical data collection across multiple pandemic periods, along with linkage to the national testing database, reduced recall and selection bias, strengthening the validity of study findings.

Several limitations should be considered. Using an area-level measure of deprivation limits individual-level conclusions, and categorising migration and ethnic minority status as binary variables, while necessary for adequate sample sizes, restricted a more in-depth analysis of PCC risks across specific migrant groups and ethnicities. Future studies should explore these differences more granularly.

The non-probability sampling design of the Virus Watch cohort introduced potential selection bias. Certain demographic and socioeconomic groups, specifically deprived, migrant and ethnic minority groups, were more likely to be under-represented due to language barriers, limited internet access and economic circumstances. This may lead to an underestimation of PCC risk in these populations, restricting the generalisability of our findings.^43^ Additionally, restricting non-PCC participants to those who have completed all four long-term symptom surveys helped minimise misclassification bias but may have disproportionately excluded individuals from deprived backgrounds, migrants and ethnic minorities, who may face barriers to consistent survey participation. In contrast, as PCC cases were identified from any of the four surveys, this may have inflated the proportion of PCC cases relative to non-PCC cases. However, applying the same restriction to PCC cases would significantly reduce the sample size and statistical power. Therefore, these selection criteria were used as they optimised the balance between ensuring the validity of the non-PCC cohort and maintaining statistical power for the analysis.

Finally, misclassification of PCC cases may have arisen from limiting community testing, recall bias and missed asymptomatic infections, particularly during the pre-Omicron (Wild Type and Alpha) periods.

## Conclusion

Our findings underscore the critical need to tackle health inequalities to minimise the infection risk and long-term consequences of COVID-19 and future pandemics, especially for deprived, migrant and ethnic minority groups. This includes addressing preinfection determinants such as mitigating occupational and housing-related exposure risks. Targeted policies and interventions should focus on the specific needs of these groups in vaccination, recovery and rehabilitation efforts. Early efforts to increase vaccine uptake and ensure equitable access to PCC and primary care services in these communities are crucial to reducing their infection and subsequent PCC risk. Providing culturally and linguistically appropriate care and collaborating with trusted community partners is also necessary to disseminate accurate public health information about PCC. Further investigation into the intersectionality of multiple social dimensions, including sex and occupation, on PCC risk is recommended to guide targeted public health efforts and resource allocation for the most vulnerable populations.

## Supplementary material

10.1136/jech-2024-223491 online supplemental file 1

## Data Availability

Data are available upon reasonable request.
